# Efficacy of a Single-Session “Empowered Relief” Zoom-Delivered Group Intervention for Chronic Pain: Randomized Controlled Trial Conducted During the COVID-19 Pandemic

**DOI:** 10.2196/29672

**Published:** 2021-09-10

**Authors:** Maisa S Ziadni, Lluvia Gonzalez-Castro, Steven Anderson, Parthasarathy Krishnamurthy, Beth D Darnall

**Affiliations:** 1 Department of Anesthesiology, Perioperative and Pain Medicine Stanford University School of Medicine Palo Alto, CA United States; 2 C.T. Bauer College of Business University of Houston Houston, TX United States

**Keywords:** single-session, empowered relief, Zoom-delivered, pain catastrophizing, pain intensity, randomized-controlled trial, chronic pain

## Abstract

**Background:**

Cognitive behavioral therapy–pain is an evidence-based treatment for chronic pain that can have significant patient burden, including health care cost, travel, multiple sessions, and lack of access in remote areas.

**Objective:**

The study aims to pilot test the efficacy of a single-session videoconference-delivered *empowered relief* (ER) intervention compared to waitlist control (WLC) conditions among individuals with chronic pain. We hypothesized that ER would be superior to WLC in reducing pain catastrophizing, pain intensity, and other pain-related outcomes at 1-3 months posttreatment.

**Methods:**

We conducted a randomized controlled trial involving a web-based sample of adults (N=104) aged 18-80 years with self-reported chronic pain. Participants were randomized (1:1) to 1 of 2 unblinded study groups: ER (50/104, 48.1%) and WLC (54/104, 51.9%). Participants allocated to ER completed a Zoom-delivered class, and all participants completed follow-up surveys at 2 weeks and 1, 2, and 3 months posttreatment. All the study procedures were performed remotely and electronically. The primary outcome was pain catastrophizing 1-month posttreatment, with pain intensity, pain bothersomeness, and sleep disruption as secondary outcomes. We also report a more rigorous test of the durability of treatment effects at 3 months posttreatment. Data were collected from September 2020 to February 2021 and analyzed using intention-to-treat analysis. The analytic data set included participants (18/101, 17.8% clinic patients; 83/101, 82.1% community) who completed at least one study survey: ER (50/101, 49.5%) and WLC (51/104, 49%).

**Results:**

Participants (N=101) were 69.3% (70/101) female, with a mean age of 49.76 years (SD 13.90; range 24-78); 32.7% (33/101) had an undergraduate degree and self-reported chronic pain for 3 months. Participants reported high engagement (47/50, 94%), high satisfaction with ER (mean 8.26, SD 1.57; range 0-10), and high satisfaction with the Zoom platform (46/50, 92%). For the between-groups factor, ER was superior to WLC for all primary and secondary outcomes at 3 months posttreatment (highest *P*<.001), and between-groups Cohen *d* effect sizes ranged from 0.45 to 0.79, indicating that the superiority was of moderate to substantial clinical importance. At 3 months, clinically meaningful pain catastrophizing scale (PCS) reductions were found for ER but not for WLC (ER: PCS −8.72, 42.25% reduction; WLC: PCS −2.25, 11.13% reduction). ER resulted in significant improvements in pain intensity, sleep disturbance, and clinical improvements in pain bothersomeness.

**Conclusions:**

Zoom-delivered ER had high participant satisfaction and very high engagement. Among adults with chronic pain, this single-session, Zoom-delivered, skills-based pain class resulted in clinically significant improvement across a range of pain-related outcomes that was sustained at 3 months. Web-based delivery of ER could allow greater accessibility of home-based pain treatment and could address the inconveniences and barriers faced by patients when attempting to receive in-person care.

**Trial Registration:**

ClinicalTrials.gov NCT04546685; https://clinicaltrials.gov/ct2/show/NCT04546685

## Introduction

### Background

Chronic pain is a significant public health concern. It is one of the most common reasons for seeking medical care [1], with considerable societal and financial burden [2,3] in addition to human suffering. Chronic pain treatment is challenging due to limitations of existing pharmacological approaches, particularly intolerability of many medicines [4], health risks [5-9], and lack of sustainable availability. In contrast, behavioral medicine approaches are low risk and have been shown to have low-to-moderate efficacy for a range of chronic pain conditions [5,7,10-12], with cognitive behavioral therapy (CBT)–pain as the established evidence-based treatment for chronic pain [8,9]. However, several barriers prevent broad access to CBT-pain, including out-of-pocket costs, burdensome travel, lack of availability in remote or rural areas, and a lack of adequately trained clinicians [6,13]. The length of treatment (ie, eight sessions or more for CBT-pain) is associated with increased dropout [14], which may perpetuate the overuse of medical services and health disparities. The COVID-19 pandemic has further challenged the feasibility of multisession in-person treatment options [15], and this creates the need for accessible solutions that are low-cost, low-burden, and remove the need for face-to-face meetings [16].

Brief web-based and remotely delivered psychological interventions have been demonstrated to accomplish this goal and have been found to be as effective as face-to-face therapy, while decreasing health care use and barriers to treatment [17]. Effective web-based treatments now exist for various psychological disorders, including depression, panic disorder, and social phobia [18]. Web-based CBT has been effective for a number of chronic health conditions, including irritable bowel syndrome, tinnitus, and headache [19,20]. In a systematic review of web-based interventions for chronic back pain, Garg et al [21] reported that web-based interventions, including CBT, effectively reduced pain catastrophizing scale (PCS) scores. Effect sizes for web-based interventions for chronic pain have been estimated to range from small to moderate (Cohen *d* range 0.04-1.23) [18]. In addition to CBT, existing web-based interventions for patients with pain include compassionate mind training [22], social media–based web-based community intervention [23], pain self-management [24], and hypnosis [25]. Despite the promise of web-based interventions for pain, multisession treatments typically range from 20 days to 12 weeks in duration, which can be costly, burdensome, and limit access to adequate care [26].

Single-session interventions (SSIs), which have been defined as the intentional delivery of a specific, structured program involving a single visit with a clinic, provider, or program [27], have the potential to reduce the burden of traditional multisession treatment approaches and expand access to behavioral medicine. SSIs have been found to be effective for a variety of psychological and health conditions, including severe mental illness [28], anxiety and youth conduct disorder [27], acute insomnia [29], heavy alcohol consumption in college students [30], trauma and adversity [31,32], postsurgical pain [33], and chronic pain [11,12,34-36]. SSIs that have been further optimized via web-based delivery have demonstrated feasibility and efficacy similar to face-to-face interventions [11,12,37] and include interventions for multiple sclerosis pain [38], disordered gambling [39], and adolescent mental health [40-42]. In addition to reducing treatment burdens, web-based SSIs have the vital advantage of ease of scalability, as they can be completed by patients in any location [37]. Effective web and digital-based SSIs would eliminate or reduce many of the existing barriers to treatment, such as cost, lack of trained therapists, and insurance limits on the length of treatment. However, despite the promise of SSIs, rigorously designed and randomized controlled studies are needed to establish their efficacy [43].

### Objectives

Building on the limited literature on web-based SSIs for chronic pain, we aim to evaluate a Zoom-delivered version of a previously developed and efficacious, skills-based, behavioral medicine SSI *empowered relief* (ER) [11,36]. Previously, our group conducted a three-arm randomized controlled trial conducted in 263 adults with chronic low back pain comparing in-person ER to a health education class or 8-week CBT [12]. However, web-based delivery of ER has not been tested, particularly in mixed-etiology chronic pain, and the COVID-19 context underscores the importance of providing effective home-based chronic pain care. Extending this work to the digital platform, in this study, we conducted a parallel-group, randomized (1:1) comparative efficacy trial to assess the impact of a single-session videoconference-delivered ER group pain relief skills class versus waitlist control (WLC). We hypothesize that at 3 months posttreatment, (1) ER would be superior to WLC for reductions in pain catastrophizing, an index of pain coping; (2) ER would be superior to WLC for reductions in pain intensity, bothersomeness, and sleep disturbance; and (3) ER would be superior to WLC in reducing anxiety, depression, and physical function.

## Methods

### Overview and Setting

This study was a parallel-group, randomized, clinical trial comparing ER, a 2-hour single-session videoconference-delivered class, to a WLC. Enrolled participants (N=104) aged 18-80 years with mixed chronic pain etiology were randomized (1:1) to one of the two study arms. Participants completed outcome assessments at pretreatment, week 2, and 1, 2, and 3 months posttreatment. The primary outcome was pain catastrophizing levels at the 1-month follow-up [35], with pain intensity, pain, bothersomeness, and sleep disturbance as secondary outcomes. The tertiary outcomes included PROMIS (Patient-Reported Outcomes Measurement Information System) measures of anxiety, depression, and physical function at the 1-month follow-up time-point. We also report a more rigorous test of the durability of treatment effects at 3 months posttreatment as highlighted in the *Results* section. A crossover intervention class was offered to participants who were initially randomized to the WLC and was conducted in February 2021. Of 48 participants who were offered the treatment, 20 (42%) attended the crossover class at no cost, and no data were collected. This study was conducted between August 2020 and February 2021.

### Study Sample, Recruitment, and Participant Compensation

**Figure 1:**
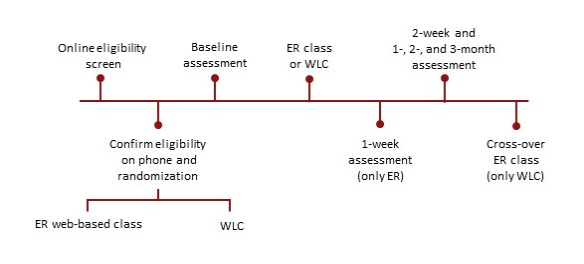
Participant activities. ER: empowered relief; WLC: waitlist control.

Participants were recruited remotely through targeted emails to lists of patients who agreed to be contacted for research purposes through two databases: (1) the Stanford’s Collaborative Health Outcomes Information Registry comprising patients who have received care at the Stanford Pain Management Center (a tertiary referral pain clinic in Redwood City, California) and (2) the Stanford Systems Neuroscience and Pain Lab database of individuals with chronic pain. Interested individuals were directed to a web-based screening form, and those who met the initial eligibility criteria were contacted by research staff by phone to confirm their eligibility. Approximately 18.3% (19/104) of the study sample were clinic patients, and 81.7% (85/104) were from the larger community. Eligible and interested participants were invited to participate in a pain-skills treatment class compared with a WLC and completed an electronic informed consent form (see Multimedia Appendix 1 for the study consent form). After informed consent was obtained, study participants were randomized one-to-one using a REDCap (Research Electronic Data Capture; Vanderbilt University) [44] cloud random number generator and allocated to the study group. All study procedures were completed remotely, and no in-person visits were required. Figure 1 displays the participants’ study activities.

All the study procedures were performed remotely and electronically. Enrolled participants completed a baseline survey, which was conducted 4 days before the scheduled class, followed by posttreatment surveys at 2 weeks, and 1, 2, and 3 months. The intervention was conducted on Zoom [45], and details of the Zoom session were emailed to participants before the scheduled class time. Overall, in the ER arm, two cohorts were recruited, each consisting of 24-26 participants per class. The class sizes were consistent with studies on web-based interventions [46-51]. Participants received US $10 in the form of an Amazon electronic gift code for each survey they completed and could receive up to US $50 following completion of all study procedures. The research procedures were approved by the Stanford institutional review board.

### Inclusion and Exclusion Criteria

All study recruitment efforts directed interested individuals with chronic pain to the web-based screening form to complete an automated eligibility form that screened for initial inclusion and exclusion criteria. Participants who met initial eligibility were then contacted by the research staff to confirm study eligibility according to the inclusion and exclusion criteria provided in Textbox 1. Internet and computer literacy were the implicit de facto eligibility criteria. If interested participants indicated that they had never used the Zoom platform, the research coordinator contacted them by phone to schedule an individual meeting; their skill to participate in the web-based class would increase because they were more familiar with the Zoom platform and navigating the tool in a supervised setting. Of the 9 participants who indicated that they had never used Zoom, none requested to schedule an individual meeting.

**Inclusion and exclusion criteria.**

**Inclusion criteria**

- Pain >3 months more than half the time
- English fluency
- Ability to attend a one-time web-based class (if assigned) and complete web-based surveys
- Females and males, aged ≥18 years

**Exclusion criteria**

- Ongoing legal action or disability claim
- Previous participation in the empowered relief class
- Cognitive impairment, non–English-speaking, or psychological factors that would preclude comprehension of material or full participation in the study

### Randomization and Blinding

Participants were randomized 1:1 (no blocking applied) and allocated to one of two study arms: (1) ER or (2) WLC. REDCap was used to apply an automatic and blinded randomization program and ensure equal allocation to both groups. Participants were not blinded to the study arm, given the nature of WLC. Statisticians performed blinded analysis of data sets that were randomly labeled as group 1 and group 2, with statistician unblinding occurring only after posttreatment month 3 data were analyzed. The study coordinator (LGC) was unblinded to the individual study arm assignments and was not involved in any data analyses. The study protocol was reviewed and approved [52].

### Study Groups

#### Single-Session Videoconference-Delivered Skills-Based Pain Class (ER)

##### Overview

ER was developed in 2013, with pilot data [36] showing a reduction in pain catastrophizing 1-month posttreatment, despite comorbid emotional disorders. Notably, a National Institute of Health-funded, three-arm, randomized controlled trial [11,12] conducted in 263 individuals with chronic low back pain demonstrated that 3-month posttreatment, the ER class was equally effective to an 8-week CBT-pain intervention and was superior to a health education control class in reducing pain catastrophizing and pain intensity. ER *compresses* key elements and skills from CBT-pain and mindfulness into a single-session 2-hour class [11,12,34,36]. The class is didactic and delivered with a standardized instructor slide deck and manual. Participants learn self-management skills to decrease physiological hyperarousal, which includes diaphragmatic breathing, cognitive reframing, and self-soothing strategies. During the class, participants self-tailor the skills content by completing a personalized plan for ER. They also received an MP3 audio file with a 20-minute version of the relaxed breathing exercise. The participants can download the audio file from a secure web-based storage platform onto their various devices (mobile or tablet) for convenience. The participants were encouraged to practice the learned skills daily.

##### Class Platform

The Zoom platform was used to deliver the ER classes, and instructors shared their PowerPoint presentation slides throughout the class. Class participants were encouraged to ask questions at any time through the Zoom chat box and unmute themselves to participate. Zoom is password-protected and hosted within the firewalled Stanford University School of Medicine and Stanford HealthCare systems.

##### Instructors

The class instructors were doctoral-level clinical psychologists certified by Stanford University [53] to deliver the intervention.

##### Training and Monitoring of Instructors

The instructors were familiar with the study protocol, were certified in ER delivery, and had extensive experience delivering the class. ER was developed to ensure fidelity (eg, standardized slide deck and instructor manual; instructor certification process), and cohort effects were minimal due to the single session and didactic nature of the intervention, with minimal participant interaction.

#### Waitlist Control

The study used a WLC group because the ER class web-based delivery is a new approach that has not been previously tested [54,55]. During the screening phone call, participants randomized to the WLC were advised to continue any ongoing clinical care. Although the WLC group did not receive any study intervention, participants were informed that they would be offered the ER treatment class after study completion at 3 months.

### Data Collection and Variable Measurement

All surveys were collected through REDCap [44], a web-based electronic data capture platform, which is a secure (password protected), HIPAA (Health Insurance Portability and Accountability Act)–compliant platform, and hosted by the Stanford University School of Medicine.

Data collection consisted of electronically collected participant-reported measures across the four phases of the study: screening, pretreatment, treatment, and posttreatment. For both groups, posttreatment survey collection was conducted at 2 weeks and 1-3 months posttreatment. The ER group completed an additional brief survey 1-week posttreatment to assess participant satisfaction with the intervention and Zoom platform. Table 1 shows the data collection time-points for both the groups. Baseline surveys included demographic information, pain intensity, pain bothersomeness, pain catastrophizing, and PROMIS measures. Posttreatment surveys included the same measures, excluding the demographics.

**Table 1:** Timeline of variable assessment.

Variables		Pretreatment	Posttreatment follow-up		
		Baseline	1 week	2 weeks	1, 2, and 3 months
Demographics and pain condition		✓^a^			
PCS^b^		✓		✓	✓
Pain bothersomeness		✓		✓	✓
**PROMIS^c^**					
	Depression	✓		✓	✓
	Anxiety	✓		✓	✓
	Pain intensity	✓		✓	✓
	Physical function	✓		✓	✓
	Pain interference	✓		✓	✓
	Fatigue	✓		✓	✓
	Social isolation	✓		✓	✓
	Anger	✓		✓	✓
	Sleep disturbance	✓		✓	✓
Satisfaction with treatment			✓		

^a^Variable assessed.

^b^PCS: pain catastrophizing scale.

^c^PROMIS: Patient-Reported Outcomes Measurement Information System.

### Study Variables and Measures

#### Pain Catastrophizing

The 13-item PCS [56] measures negative thoughts and emotional responses to pain. PCS is a 1D measurement with three subscales: helplessness, magnification, and rumination. It is scored by summing all items and generates a total score, with higher scores indicating greater catastrophizing. The PCS is a reliable, validated, and psychometrically trusted instrument [57].

#### Pain Bothersomeness

Participants rated their pain bothersomeness during the previous 7 days on a 0-10 numeric rating scale anchored by 0 (not at all bothersome) and 10 (extremely bothersome) that is commonly used in chronic low back pain research [58], “How bothersome has your pain been during the past week?”

#### National Institutes of Health PROMIS Measures

The National Institutes of Health PROMIS short-form measures have been applied in pain research [59-67], and selected domains were identified by the Initiative on Methods, Measurement, and Pain Assessment in Clinical Trials [68,69] as core outcomes. Respondents reference the previous 7 days to rate items for pain intensity (version 3a), sleep disturbance (version 6a), physical function (version 6b), depression (version 6a), anxiety (version 6a), and social isolation (4a). Higher scores on PROMIS measures indicate greater symptom severity, except for physical function, wherein higher scores reflect better function. The web-based PROMIS assessment center [70] software [71] was used to calculate the short-form T scores using Item Response Theory scoring algorithms that apply the Bayesian expected A posteriori method [72]. Depression, anxiety, and physical function are major correlates of chronic pain [73-76], and as such, changes in these variables were examined as a function of treatment. Social isolation was included because of the COVID-19 pandemic. We did not expect it to change, per se, but given the dynamic nature of COVID-19 and lockdowns, we measured this variable throughout the study.

### Sample Size and Statistical Analysis

The planned enrollment accounted for 4% attrition in each study arm. The enrolled sample size ensured significant statistical power (80%) to detect medium-to-large treatment effects between the two treatment groups. Of the 104 enrolled participants (ER: 50/104, 48.1%; WLC: 54/104, 51.9%), intent-to-treat analyses were used to analyze the data of 101 (97.1%) participants (ER: 50/101, 49.5%; WLC: 51/101, 50.5%) who met the study criteria and completed at least one survey.

Baseline scores on continuous variables were summarized using mean (SD), and categorical variable scores were summarized by count and proportions. The differences between conditions at baseline for continuous and categorical variables for the treatment groups were assessed using one-way analysis of variance and Fisher exact test, respectively. For primary analyses, we used a between-within mixed design to assess the effect of treatment, time, and treatment × time, where time represented the within-subjects factor and treatment was specified as a between-subjects factor. Within-subjects’ dependency was modeled by specifying participants as a random effect with a heterogeneous compound symmetry covariance structure. Similar analyses were used to examine the treatment effects for all secondary and tertiary outcomes at each posttreatment month. Intention-to-treat analysis was used to investigate the causal effects of the treatment. All statistical significances were applied at a two-sided level of 0.05, with Benjamini-Hochberg adjustment for multiple comparisons. Cohen *d* effect sizes for the between-subjects’ factor were calculated from the between-subjects’ *F* statistic adjusting for sample size. Responder analyses were conducted by calculating the proportion of participants with 15% (minimal), 30% (moderate), and 50% (substantial) improvement from baseline for pain-related outcomes. All data processing and statistical analyses were performed using the SAS version 9.4 (SAS Institute, Inc).

## Results

### Study Participants

**Figure 2:**
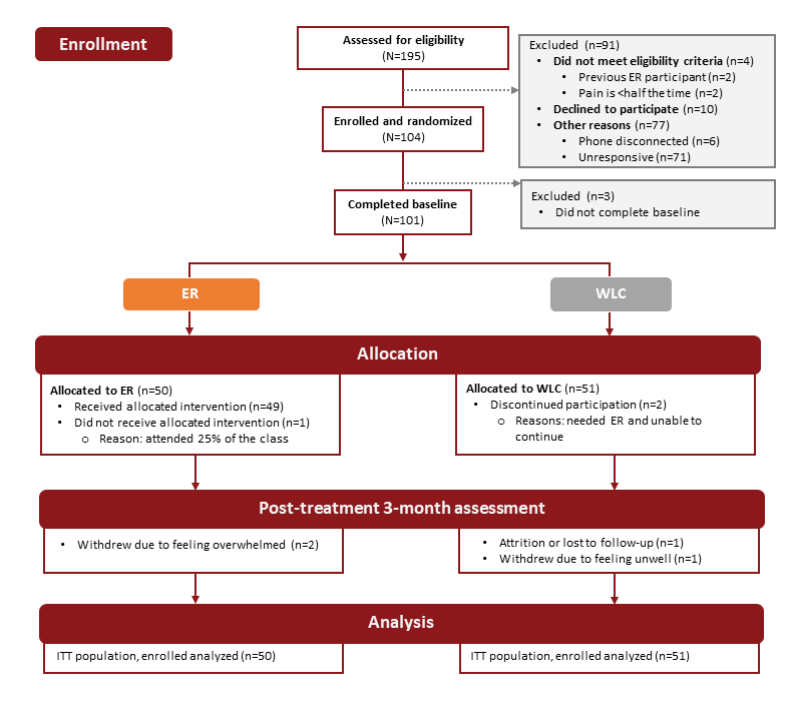
CONSORT (Consolidated Standards of Reporting Trials) flow diagram. ER: empowered relief; ITT: intention-to-treat; WLC: waitlist control.

Figure 2 shows the CONSORT (Consolidated Standards of Reporting Trials) diagram for the study. In total, 196 individuals were assessed for initial eligibility, and 92 were excluded because they did not meet the study criteria. In total, 104 participants were enrolled and randomized, and 101 (97.1%) participants completed the baseline surveys (ER: 50/101, 49.5%; WLC: 51/101, 50.5%). A total of 49 participants received the ER intervention, of whom 2 (4%) were lost to follow-up due to feeling unwell or being too busy to complete follow-up surveys. A final sample of 94 participants provided complete follow-up data. Nearly 94% (47/50) of participants in the ER group and 86% (44/51) of participants in the WLC group completed their 3-month follow-up surveys (end of study).

Table 2 shows the baseline demographic characteristics of the study arm. The sample included 101 participants recruited primarily from northern California. The sample was predominantly female (70/101, 69.3%), White (75/101, 74.3%), and with at least some college education (66/101, 65.3%), and a mean age of 49.76 years (SD 13.90; range 24.00-78.00). No significant between-group differences were observed for any demographic variables, demonstrating that randomization was effective.

**Table 2:** Baseline demographic characteristics by treatment group.

Demographics			Values					*P* value
			Treatment group with empowered relief (n=50)		WLC^a^ (n=51)			
**Gender, n (%)**								.15^b^
	Male	12 (24)		19 (37)				
	Female	38 (76)		32 (63)				
**Age (years)**								.34^c^
	Mean (SD; range)	48.6 (14.1; 26.0-78.0)		50.9 (13.7; 24.0-74.0)				
	Median (IQR)	48.5 (35.0-59.0)		51.0 (40.0-67.0)				
**Race, n (%)**								.90^b^
	Asian	6 (12)		7 (14)				
	White	37 (74)		38 (75)				
	Black or African American	1 (2)		0 (0)				
	More than one race	3 (6)		3 (6)				
	Other or unknown	3 (6)		3 (6)				
**Education, n (%)**								.20^b^
	High school graduate	1 (2)		1 (2)				
	Some college	7 (14)		7 (14)				
	Associate	8 (16)		11 (22)				
	Undergraduate	14 (28)		19 (37)				
	Master’s degree	13 (26)		10 (20)				
	Professional degree	1 (2)		3 (6)				
	Doctoral degree	6 (12)		0 (0)				
**Employment, n (%)**								.72^b^
	Part-time	10 (20)		10 (20)				
	Full-time	15 (30)		18 (35)				
	Full-time homemaker	0 (0)		4 (8)				
	Temporarily laid off	3 (6)		1 (2)				
	Unemployed	3 (6)		2 (4)				
	Looking for work, unemployed	3 (6)		2 (4)				
	Disabled or not working due to pain	5 (10)		4 (8)				
	Retired	5 (10)		6 (12)				
	Student, currently employed	1 (2)		1 (2)				
	Student, not currently employed	1 (2)		0 (0)				
	Other	4 (8)		3 (6)				
**Income (US $), n (%)**								.64^b^
	<10,000	1 (2)		2 (4)				
	>25,000	4 (8)		7 (14)				
	25,000-44,999	6 (12)		3 (6)				
	45,000-64,999	2 (4)		3 (6)				
	65,000-84,999	2 (4)		4 (8)				
	85,000-104,999	7 (14)		3 (6)				
	105,000-124,999	7 (14)		10 (20)				
	>125,000	15 (30)		14 (27)				
	Missing	6 (12)		5 (10)				
**Relationship, n (%)**								.82^b^
	Never married	10 (20)		9 (18)				
	Married	26 (52)		26 (51)				
	Divorced	6 (12)		7 (14)				
	Separated	1 (2)		2 (4)				
	Widowed	0 (0)		2 (4)				
	Partnered and living together	5 (10)		4 (8)				
	In a relationship but not living together	2 (4)		1 (2)				

^a^WLC: waitlist control.

^b^Chi-square *P* value.

^c^Kruskal-Wallis *P* value.

Table 3 presents the baseline pain and clinical characteristics of the sample by the study arm. The mean pain intensity T score was 62.2 (mean 50, SD 10). No significant differences were observed between the two study groups for any of the variables assessed, except for sleep disturbance (*P*=.04), which was controlled for in the analysis.

**Table 3:** Baseline clinical variables by treatment group.

Variables					Values				*P* value^b^	
					Treatment group with empowered relief (n=50)		WLC^a^ (n=51)			
**Pain catastrophizing**										.86
		Mean (SD; range)			20.6 (10.1; 2.0-47.0)		20.2 (10.7; 2.0-43.0)			
		Median (IQR)			19.0 (13.0-28.0)		19.0 (12.0-29.0)			
**Average pain intensity**										.60
		Mean (SD; range)			62.2 (5.7; 48.0-81.9)		62.5 (7.7; 36.3-81.9)			
		Median (IQR)			62.6 (58.9-64.1)		62.6 (60.2-67.8)			
**Pain bothersomeness**										.28
		Mean (SD; range)			5.8 (2.0; 0.0-10.0)		6.3 (2.0; 2.0-10.0)			
		Median (IQR)			6.0 (4.0-7.0)		6.0 (5.0-7.0)			
**PROMIS^c^**										
	**Depression**								.95	
			Mean (SD; range)	57.3 (9.6; 38.4-73.0)		57.8 (9.3; 38.4-80.2)				
			Median (IQR)	58.2 (50.6-65.4)		56.4 (52.3-64.7)				
	**Anxiety**								.36	
			Mean (SD; range)	59.6 (8.3; 39.1-82.4)		60.4 (8.9; 39.1-79.5)				
			Median (IQR)	60.7 (52.9-64.5)		61.8 (54.4-66.1)				
	**Physical function**								.99	
			Mean (SD; range)	40.8 (6.3; 28.7-59.0)		41.1 (7.2; 24.8-59.0)				
			Median (IQR)	40.4 (36.0-45.1)		40.5 (36.7-44.1)				
	**Interference**								.82	
			Mean (SD; range)	59.7 (6.6; 41.1-76.2)		60.3 (5.4; 51.0-76.2)				
			Median (IQR)	59.5 (55.7-64.8)		60.0 (56.2-63.8)				
	**Fatigue**								.79	
			Mean (SD; range)	61.6 (8.4; 33.7-75.8)		62.4 (9.2; 33.7-75.8)				
			Median (IQR)	62.8 (57.1-66.8)		62.7 (57.1-68.8)				
	**Social isolation**								.23	
			Mean (SD; range)	53.5 (11.0; 34.8-74.2)		52.3 (9.4; 34.8-74.2)				
			Median (IQR)	56.2 (47.9-62.1)		51.7 (47.9-56.2)				
	**Anger**								.23	
			Mean (SD; range)	54.6 (9.1; 32.9-82.9)		55.5 (12.0; 32.9-77.1)				
			Median (IQR)	54.7 (47.9-60.7)		57.8 (49.8-63.8)				
	**Sleep disturbance**								.04	
			Mean (SD; range)	56.3 (9.0; 36.4-76.1)		59.5 (8.5; 36.6-76.1)				
			Median (IQR)	54.5 (51.3-61.8)		60.0 (53.9-64.7)				

^a^WLC: waitlist control.

^b^Kruskal-Wallis *P* value.

^c^PROMIS: Patient-Reported Outcomes Measurement Information System.

### Treatment Satisfaction (ER Group Only)

For satisfaction with ER treatment on a scale of 0 (completely dissatisfied) to 10 (completely satisfied), participants (n=47) reported high satisfaction with all items related to overall satisfaction with the class (mean 8.26, SD 1.57), likelihood of recommending the class (mean 8.77, SD 1.43), class relevance (mean 7.94, SD 2.03), the usefulness of the presented information (mean 8.37, SD 1.79), and likelihood of using the skills and information learned (mean 8.66, SD 1.58).

### Primary Outcomes

**Figure 3:**
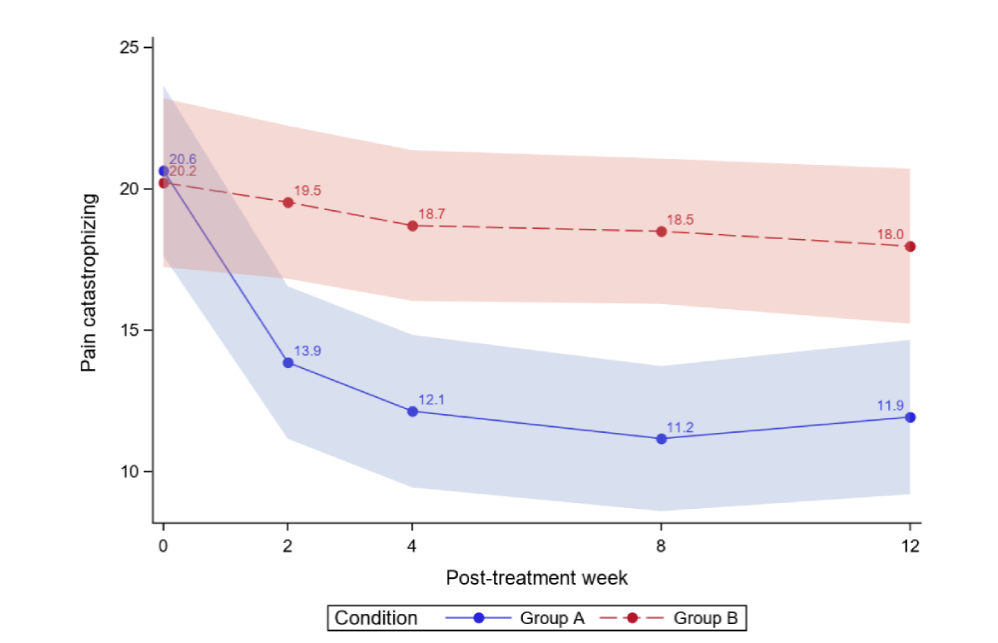
Pain catastrophizing over time.

**Figure 4:**
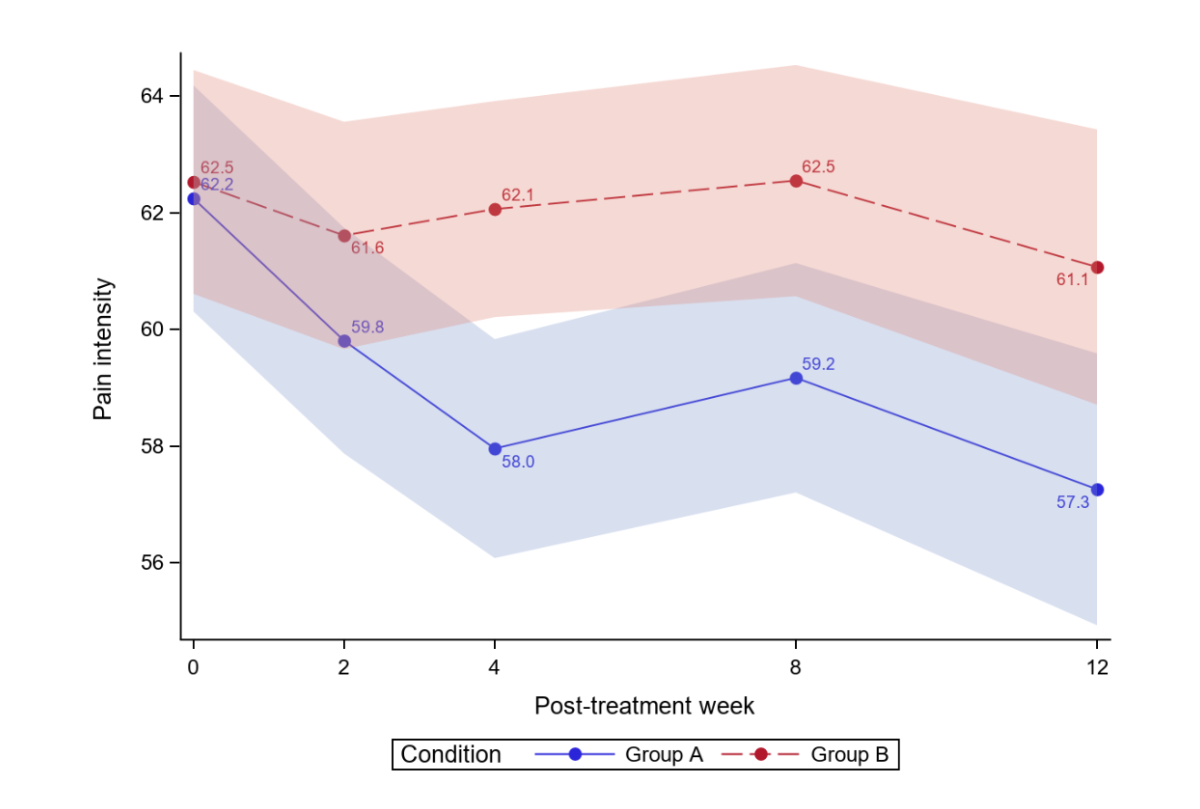
Pain intensity over time.

**Figure 5:**
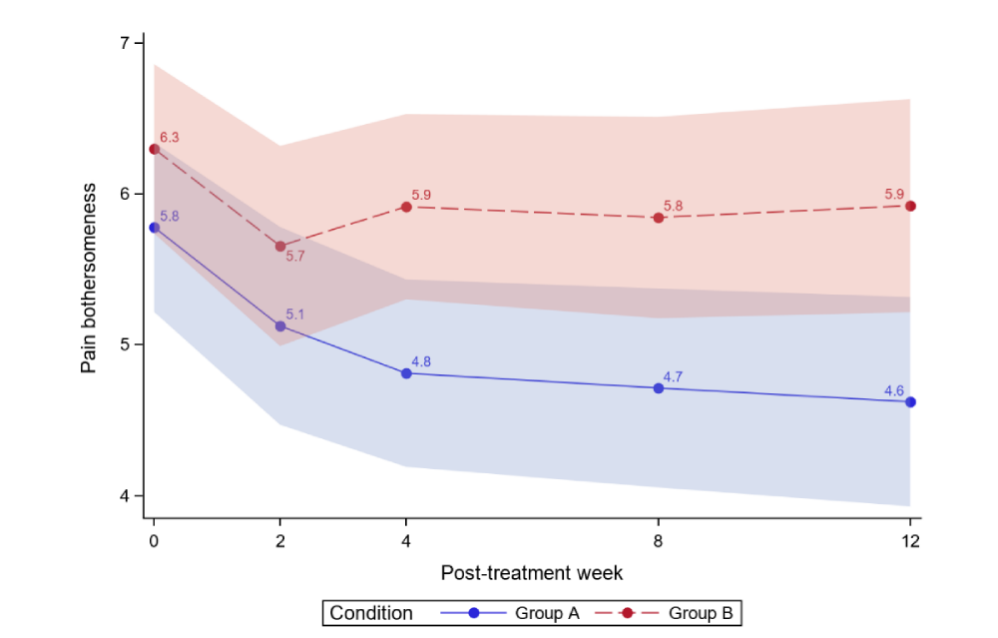
Pain bothersomeness over time.

**Figure 6:**
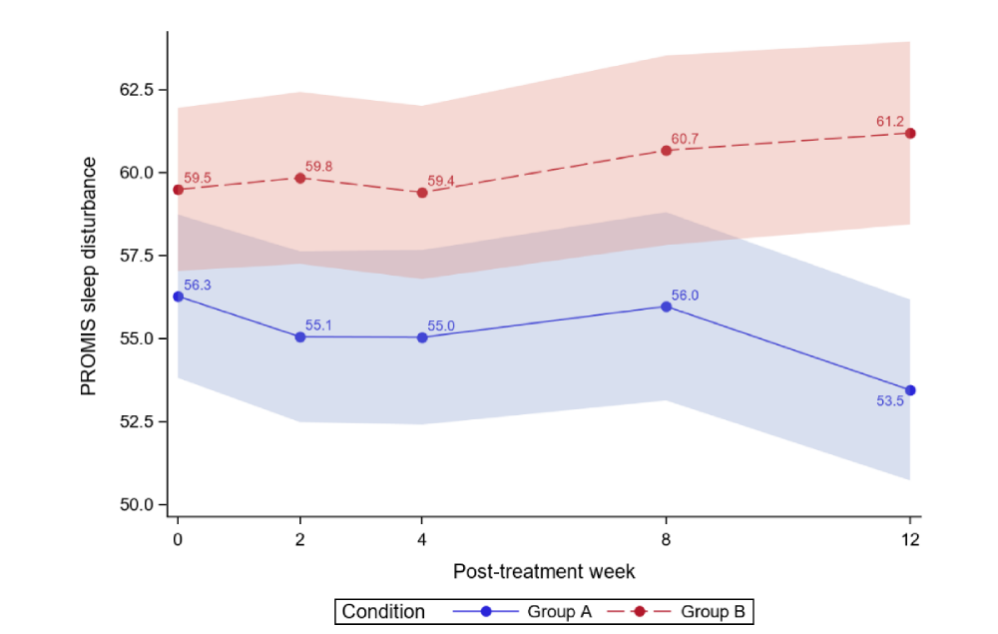
Sleep disturbance over time. PROMIS: Patient-Reported Outcomes Measurement Information System.

**Figure 7:**
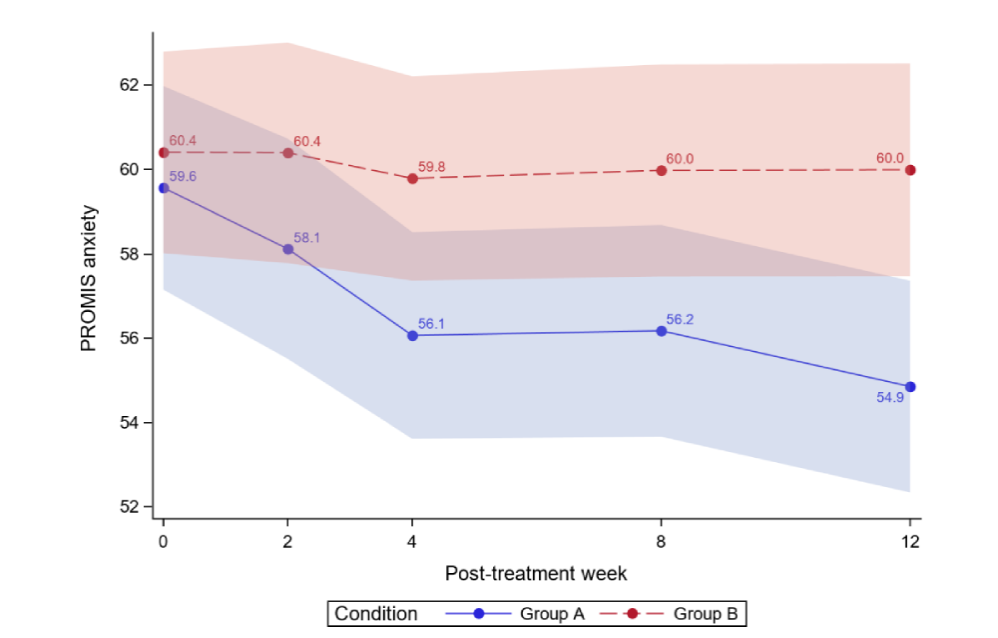
Anxiety over time. PROMIS: Patient-Reported Outcomes Measurement Information System.

**Figure 8:**
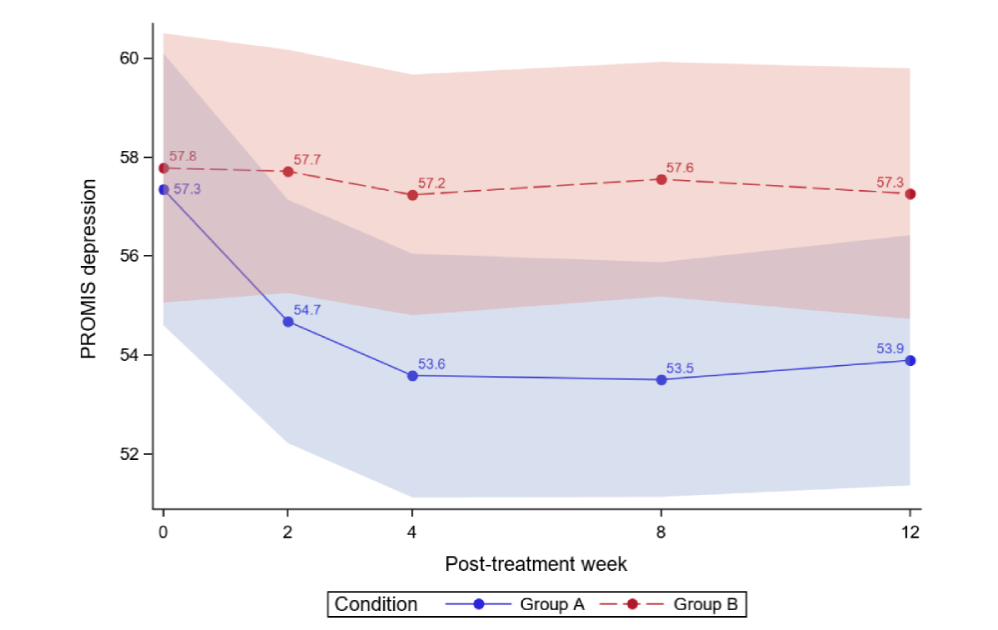
Depression over time. PROMIS: Patient-Reported Outcomes Measurement Information System.

**Figure 9:**
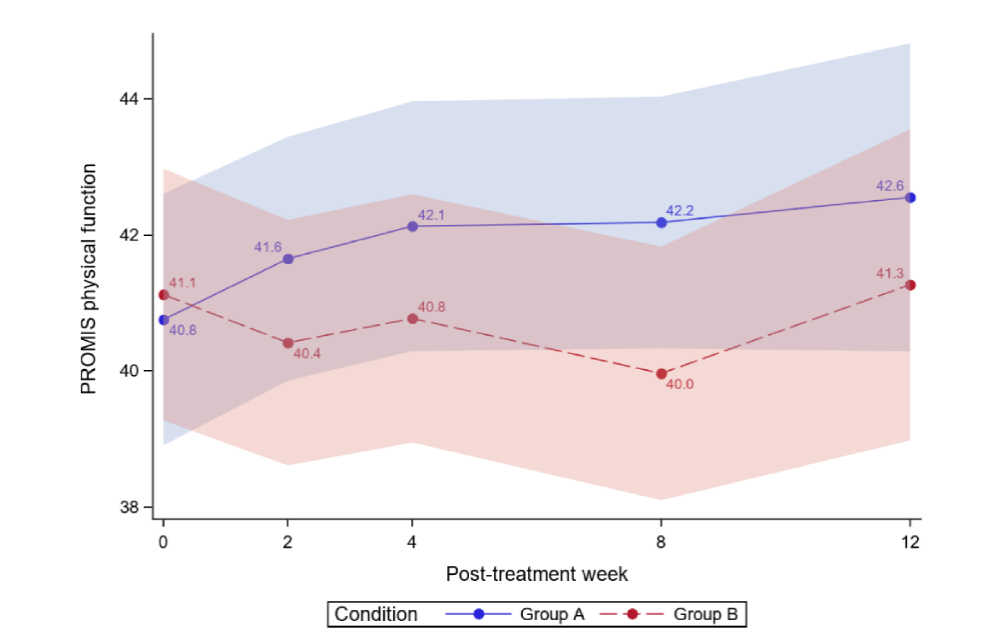
Physical function over time. PROMIS: Patient-Reported Outcomes Measurement Information System.

**Figure 10:**
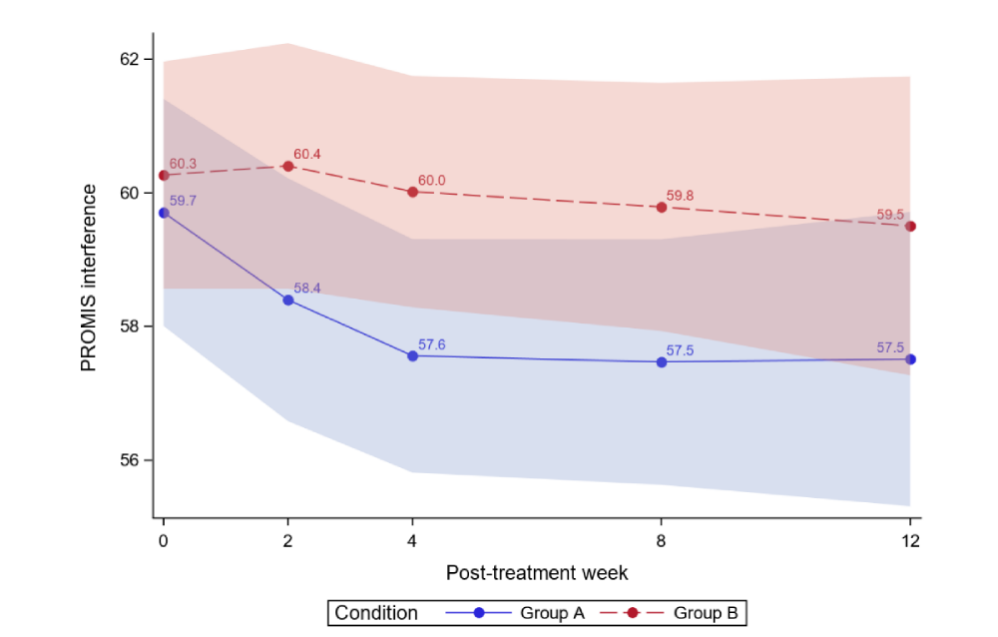
Pain interference over time. PROMIS: Patient-Reported Outcomes Measurement Information System.

**Figure 11:**
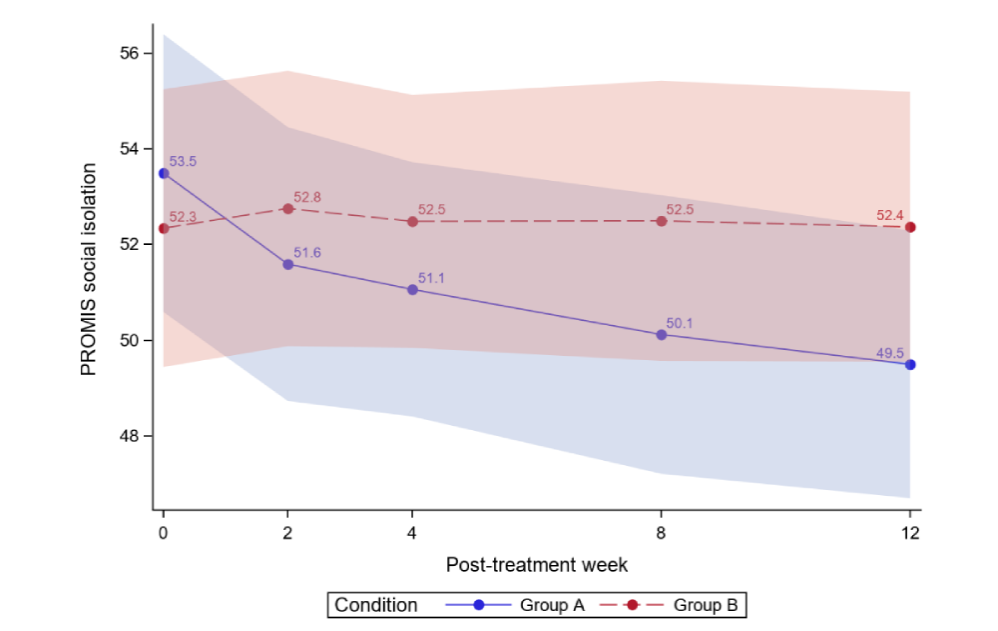
Social isolation over time. PROMIS: Patient-Reported Outcomes Measurement Information System.

**Figure 12:**
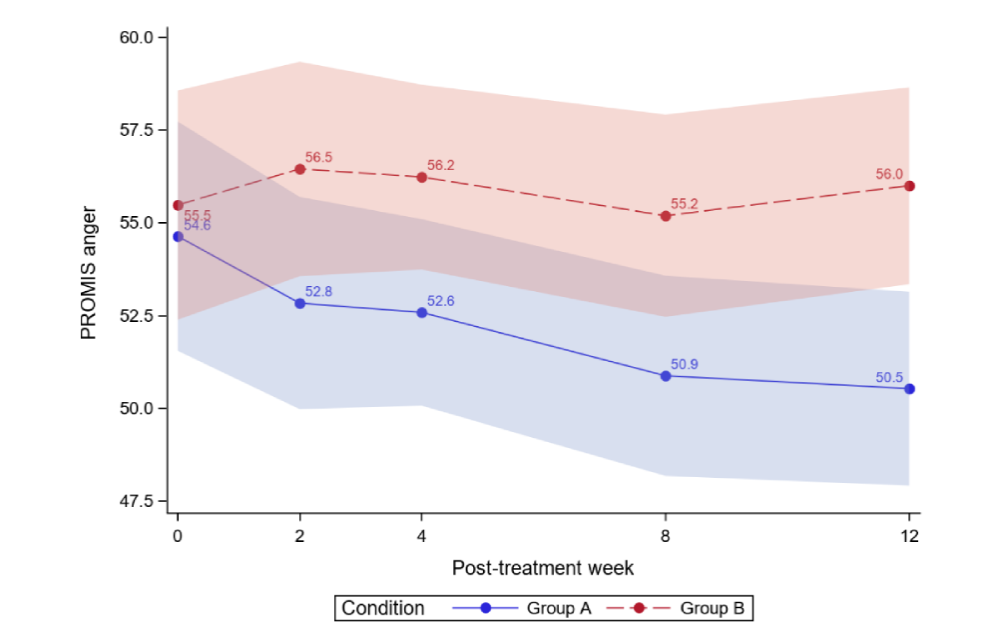
Anger over time. PROMIS: Patient-Reported Outcomes Measurement Information System.

Table 4 reports the between-group comparisons at 2 weeks and 1-3 months posttreatment for all outcomes. Figures 3-12 compare group effects over time for pain-related outcomes. For all figures, the trend of the pain-related variables is displayed over time for participants in both groups. Values in the x-axis refer to the number of weeks in the study, where 0 represents the baseline (roughly 4 days preintervention) time-point. The color band represents the 95% CI for the mean outcome variable. Overlapping bands indicate nonsignificant treatment group differences (*P* value) of simple main effects within each time-point. The corresponding model effects for each outcome are displayed in Figures 3-12.

We observed a significant treatment effect (*P*=.005) on pain catastrophizing; on average, the ER group had lower PCS scores than the WLC group (Cohen *d*=0.50). Separately, we observed a time effect; the average PCS significantly decreased over time for both study groups (time effect; *P*<.001). Most importantly, the decrease was greater for ER versus WLC (group×time effect; *P*<.001). At 3-month posttreatment, clinically meaningful reductions in PCS were found for ER but not for WLC (ER: PCS −8.72, 42.25% reduction; WLC: PCS −2.25, 11.13% reduction). Figure 3 displays the average PCS at 3 months by study arm, and Table 4 displays the between-group comparisons for PCS at baseline and posttreatment months. ER was superior to WLC (difference −6.05, 95% CI −9.92 to −2.18; *P*=.002 at 3-month posttreatment.

The ER group effect size was 0.89, with combined results showing a large effect size and moderate clinical importance. As much as 62% (31/50) of ER and 24% (12/51) of WLC participants achieved a 30% or more reduction in PCS. For ER, 46% (23/50) achieved ≥50% in PCS reduction, whereas for WLC, 12% (6/51) reached that threshold.

**Table 4:** Outcome measures from baseline to 3 months by treatment group with between-group comparisons.

Outcome measure time-point				Empowered relief, mean (SE)		WLC,^a^ mean (SE)		Between-group differences					Effect size^b^ (Cohen *d*)
								Mean difference (SE; 95% CI)		*P* value			
**Pain catastrophizing**													
	Baseline		20.64 (1.53)		20.22 (1.52)		0.42 (2.16; −3.81 to 4.66)		.84		0.04		
	2 weeks posttreatment		13.85 (1.37)		19.52 (1.38)		−5.67 (1.94; −9.49 to −1.85)		.004		0.57		
	1 month posttreatment		12.13 (1.37)		18.7 (1.35)		−6.57 (1.93; −10.36 to −2.78)		<.001		0.62		
	2 months posttreatment		11.16 (1.3)		18.5 (1.31)		−7.34 (1.85; −10.97 to −3.71)		<.001		0.76		
	3 months posttreatment		11.92 (1.39)		17.97 (1.39)		−6.05 (1.97; −9.92 to −2.18)		.002		0.64		
**Pain bothersomeness**													
	Baseline		5.78 (0.29)		6.3 (0.29)		−0.52 (0.40; −1.31 to 0.27)		.20		0.26		
	2 weeks posttreatment		5.13 (0.33)		5.66 (0.34)		−0.53 (0.47; −1.46 to 0.4)		.26		0.27		
	1 month posttreatment		4.81 (0.32)		5.92 (0.31)		−1.10 (0.44; −1.98 to −0.23)		.01		0.57		
	2 months posttreatment		4.71 (0.34)		5.84 (0.34)		−1.13 (0.48; −2.07 to −0.19)		.02		0.60		
	3 months posttreatment		4.62 (0.35)		5.92 (0.36)		−1.30 (0.50; −2.29 to −0.31)		.01		0.67		
**PROMIS^c^**													
	**Pain intensity**												
		Baseline	62.24 (0.98)		62.53 (0.97)		−0.28 (1.39; −3.01 to 2.44)		.84		0.04		
		2 weeks posttreatment	59.81 (0.98)		61.61 (0.99)		−1.8 (1.40; −4.54 to 0.94)		.20		0.27		
		1 month posttreatment	57.96 (0.95)		62.06 (0.94)		−4.1 (1.34; −6.73 to −1.47)		.002		0.62		
		2 months posttreatment	59.17 (1)		62.55 (1.01)		−3.38 (1.42; −6.17 to −0.59)		.02		0.54		
		3 months posttreatment	57.26 (1.18)		61.07 (1.2)		−3.81 (1.68; −7.12 to −0.49)		.02		0.60		
	**Physical function**												
		Baseline	40.76 (0.94)		41.12 (0.94)		−0.37 (1.33; −2.98 to 2.24)		.78		0.05		
		2 weeks posttreatment	41.65 (0.91)		40.42 (0.92)		1.23 (1.29; −1.31 to 3.77)		.34		0.24		
		1 month posttreatment	42.13 (0.93)		40.77 (0.93)		1.35 (1.31; −1.23 to 3.94)		.30		0.26		
		2 months posttreatment	42.18 (0.94)		39.97 (0.95)		2.22 (1.33; −0.41 to 4.84)		.01		0.41		
		3 months posttreatment	42.55 (1.15)		41.27 (1.16)		1.28 (1.63; −1.93 to 4.5)		.43		0.25		
	**Pain interference**												
		Baseline	59.71 (0.86)		60.27 (0.86)		−0.56 (1.22; −2.96 to 1.84)		.65		0.09		
		2 weeks posttreatment	58.4 (0.92)		60.41 (0.93)		−2 (1.31; −4.59 to 0.58)		.13		0.32		
		1 month posttreatment	57.56 (0.89)		60.02 (0.88)		−2.46 (1.25; −4.92 to 0)		.05		0.43		
		2 months posttreatment	57.47 (0.93)		59.79 (0.94)		−2.32 (1.33; −4.94 to 0.29)		.08		0.43		
		3 months posttreatment	57.51 (1.12)		59.51 (1.14)		−1.99 (1.60; −5.14 to 1.15)		.21		0.33		
	**Anxiety**												
		Baseline	59.57 (1.23)		60.41 (1.22)		−0.84 (1.73; −4.24 to 2.56)		.63		0.10		
		2 weeks posttreatment	58.12 (1.33)		60.4 (1.33)		−2.28 (1.88; −5.97 to 1.42)		.23		0.28		
		1 month posttreatment	56.06 (1.25)		59.79 (1.23)		−3.72 (1.75; −7.17 to −0.28)		.03		0.41		
		2 months posttreatment	56.18 (1.28)		59.98 (1.28)		−3.8 (1.81; −7.36 to −0.25)		.04		0.49		
		3 months posttreatment	54.85 (1.28)		59.99 (1.28)		−5.14 (1.81; −8.7 to −1.58)		.005		0.68		
	**Depression**												
		Baseline	57.35 (1.4)		57.78 (1.38)		−0.43 (1.97; −4.3 to 3.44)		.83		0.05		
		2 weeks posttreatment	54.68 (1.25)		57.71 (1.25)		−3.03 (1.77; −6.51 to 0.45)		.09		0.32		
		1 month posttreatment	53.58 (1.25)		57.24 (1.24)		−3.65 (1.76; −7.11 to −0.19)		.04		0.37		
		2 months posttreatment	53.5 (1.21)		57.55 (1.21)		−4.05 (1.71; −7.41 to −0.7)		.02		0.45		
		3 months posttreatment	53.89 (1.29)		57.26 (1.29)		−3.37 (1.82; −6.95 to 0.21)		.06		0.40		
	**Social isolation**												
		Baseline	53.49 (1.47)		52.34 (1.47)		1.15 (2.08; −2.95 to 5.25)		.58		0.11		
		2 weeks posttreatment	51.59 (1.45)		52.75 (1.46)		−1.16 (2.06; −5.21 to 2.89)		.57		0.19		
		1 month posttreatment	51.06 (1.35)		52.48 (1.34)		−1.42 (1.90; −5.16 to 2.32)		.46		0.23		
		2 months posttreatment	50.12 (1.48)		52.49 (1.49)		−2.37 (2.10; −6.5 to 1.75)		.26		0.33		
		3 months posttreatment	49.5 (1.42)		52.37 (1.43)		−2.87 (2.02; −6.84 to 1.1)		.16		0.38		
	**Anger**												
		Baseline	54.64 (1.57)		55.48 (1.57)		−0.84 (2.22; −5.21 to 3.53)		.70		0.08		
		2 weeks posttreatment	52.84 (1.45)		56.46 (1.47)		−3.62 (2.07; −7.68 to 0.45)		.08		0.34		
		1 month posttreatment	52.59 (1.28)		56.24 (1.27)		−3.65 (1.8; −7.18 to −0.11)		.04		0.35		
		2 months posttreatment	50.88 (1.37)		55.2 (1.39)		−4.31 (1.95; −8.15 to −0.48)		.03		0.44		
		3 months posttreatment	50.54 (1.33)		56 (1.35)		−5.47 (1.89; −9.19 to −1.75)		.004		0.59		

^a^WLC: waitlist control.

^b^These are between-subjects effect size within each time frame.

^c^PROMIS: Patient-Reported Outcomes Measurement Information System.

### Secondary Outcomes

#### Pain Intensity

Analyzing all data from baseline to 3 months, we observed a significant treatment effect (*P*=.02) on pain intensity; on average, the ER group had lower pain intensity scores than the WLC group (Cohen *d*=0.41). Separately, we observed a time effect; the average PCS significantly decreased over time for both study groups (time effect; *P*<.001). Most importantly, the decrease was greater for ER versus WLC (group×time effect; *P*=.01).

At 3 months, significant reductions in pain intensity were found for ER but not for WLC (ER: pain intensity −4.98, 8% reduction; WLC: pain intensity −1.46, 2.34% reduction). Figure 4 displays the average pain intensity during the 3-month follow-up by the study arm, and Table 4 displays the between-group comparisons for pain intensity at baseline and posttreatment months. At 3 months, ER was superior to WLC (difference −6.05, 95% CI −9.92 to −2.18; *P*<.001). Notably, when applying the 15% threshold for clinical meaningfulness, the reduction in pain intensity was not clinically meaningful for either group.

The ER group effect size was 0.76, with combined results showing a moderate-to-large effect size but no clinical importance. As much as 30% (15/50) of ER and 6% (3/51) of WLC participants achieved a 15% or greater reduction in pain intensity. For ER, 4% (2/50) achieved ≥30% reduction in pain intensity, whereas for WLC, 2% (1/51) reached that threshold.

#### Pain Bothersomeness

We observed a time effect; average pain bothersomeness significantly decreased over time for both study groups (time effect; *P*<.001); however, during the 1- to 3-month follow-ups, the between-group difference was significant (*P*=.01 to *P*=.02) per Table 4. This significance is largely driven by the initial decline in pain bothersomeness, which was steeper in the ER group and eventually plateaued for both groups (Figure 5). At 3 months, ER was superior to WLC (difference −1.30, 95% CI −2.29 to −0.31; *P*=.01).

At 3 months, clinically meaningful reductions in pain bothersomeness were found for ER but not for WLC (ER: pain bothersomeness −1.16, 20.07% reduction; WLC: pain bothersomeness −0.38, 6.03% reduction). This meets the threshold for moderate clinical meaningfulness. Figure 6 displays the average pain bothersomeness during the 3-month follow-up by the study arm, and Table 4 displays the between-group comparisons for pain intensity at baseline and posttreatment months.

The ER group effect size was 0.61, with combined results showing a moderate effect size and minimal clinical importance. As much as 34% (17/50) of ER and 12% (6/51) of WLC participants achieved ≥30% reduction in pain bothersomeness. For ER, 22% (11/50) achieved ≥50% in pain bothersomeness reduction, whereas for WLC, 3.9% (2/51) reached that threshold.

#### Sleep Disturbance

At 3 months, significant reductions in sleep disturbance were found for ER but not for WLC (ER: 2.82, 5.01% reduction; WLC: 1.7, 2.86% increase; Table 4). Separately, we did not observe a time effect (*P*=.52), and we observed a significant interaction effect (*P*=.02). Sleep disturbance was lower in the ER group than in the WLC group (*P*=.02). At 3 months, ER was superior to WLC (difference −7.74, 95% CI −11.61 to −3.87; *P*<.001). The ER group effect size was 0.37, with combined results showing a small-to-moderate effect size and no clinical importance. As much as 12% (6/50) of ER and 8% (4/51) of WLC participants achieved a 15% or more reduction in sleep disturbance.

The Benjamini-Hochberg adjustment for multiple comparisons was applied across primary and secondary variables, and statistical significance was maintained (*P*=.02 to *P*<.001).

### Tertiary Outcomes

#### Anxiety

At 3 months, significant reductions in anxiety were found for ER but not for WLC (ER: −4.71, 7.91% reduction; WLC: −0.42, 0.7% reduction; Table 4). Anxiety decreased over time in both groups, and the decline was significantly steeper in the ER group (*P*=.009). ER was superior to WLC (difference −5.14, 95% CI −8.7 to −1.58; *P*<.001). The ER group effect size was 0.59, with combined results showing a moderate effect size and no clinical importance.

#### Depression

We did not observe a treatment effect (*P*=.08) on depression. Separately, we observed a time effect; average depression significantly decreased over time for both study groups (time effect; *P*=.002). Most importantly, the decrease was greater for ER versus WLC (group×time effect; *P*=.03). However, between-group differences showed that ER was superior to WLC only during months 1 and 2 (*P*=.04 and *P*=.02, respectively). The overall interaction effect appears primarily driven by these two time-points and maintains the trend and directionality, but not statistically significant, at the 3-month follow-up (*P*=.07).

#### Physical Function

We did not observe a treatment effect (*P*=.36) or a time effect (*P*=.36) on physical function. We observed a trend for the interaction effect (*P*=.09). Between-subjects analysis revealed no significant differences at any of the follow-up time-points (*P*=.30 to *P*=.43), except at 2-months posttreatment (*P*=.01).

### Exploratory Outcomes

For social isolation, we observed a time effect; average social isolation scores significantly decreased over time for both study groups (time effect; *P*=.02). Most importantly, this was qualified by a significant group×time interaction (*P*=.02); there was a decrease in social isolation in the ER group but not in the WLC group. No time or interaction effects were observed for pain interference or anger.

### Zoom Platform Satisfaction

Participants in the ER arm provided feedback on their experience using the Zoom platform on a scale from 1 (strongly disagree) to 7 (strongly agree), with higher scores indicating a higher endorsement of each item. Overall, participants reported high satisfaction with the platform, including the ease of operating Zoom (46/50, 92% satisfaction: mean 6.45, SD 1.43), engaging with class material (mean 6.02, SD 1.45), comfort in engaging with Zoom instructor and participants (mean 5.68, SD 1.45), and feeling connected to the instructor (mean 5.43, SD 1.64).

## Discussion

### Principal Findings

We conducted the first Zoom-delivered randomized controlled trial of the group-based, SSI *ER* in a sample of individuals with mixed-etiology chronic pain. The primary goal of this study was to evaluate the feasibility, acceptability, and engagement of a single-session Zoom-delivered intervention for chronic pain. We also aimed to determine the preliminary efficacy of Zoom-delivered ER for reducing average pain intensity, pain catastrophizing, pain bothersomeness, and sleep disruption at 1-month posttreatment. Secondary and tertiary pain-related outcomes included physical function, anxiety, depression, and physical function. We also report a more rigorous test of the durability of treatment effects at 3-month posttreatment, as well as report the clinical meaningfulness of effects.

ER demonstrated high feasibility as indexed by high enrollment rate and excellent engagement; 94% (47/50) of participants in the ER group attended the class and completed all surveys through the 3-month duration of the study. The enrollment rate was 53.1% (104/196), which is five times higher than that observed in other studies [12,77] and stands as an index of both the inclusivity of the study protocol (few exclusionary criteria; all types of chronic pain) and the convenience of a single-session pain treatment study. Importantly, participants reported high overall satisfaction with the class (mean 8.26-8.77), the usefulness of presented information, and the likelihood of using the skills and information learned. In addition, participants reported high satisfaction with operating the Zoom platform (46/50, 92% satisfaction), in addition to high satisfaction with engaging with class material, other attendees, and the class instructor. Importantly, of the 9 participants who indicated they had never used the Zoom platform during screening, none requested additional or individual training with the platform. These metrics indicate superior feasibility, accessibility, and engagement with the Zoom-delivered ER intervention.

With respect to preliminary efficacy, time effects showed reductions in pain intensity and most pain-related indices, except for sleep disruption. ER demonstrated superior treatment effects on average pain intensity and pain-related catastrophizing, pain bothersomeness, and sleep disruption over the 3 months following treatment. The between-groups Cohen *d* effect sizes at 3-month posttreatment ranged from 0.60 to 0.91 for primary and secondary variables. For ER, pre- or posttreatment Cohen *d* effect sizes ranged from 0.37 to 0.89, demonstrating no clinical importance to moderate clinical importance for reducing pain-related catastrophizing, bothersomeness, and sleep disruption at the end of treatment. The treatment effect sizes across the primary and secondary outcomes suggested that a single-session, digital, skills-based intervention may have clinically meaningful effects on patient-reported outcomes. The durability of the treatment effects was demonstrated at the 3-month follow-up time-point. A greater proportion of participants in the ER group exceeded the thresholds for the clinical importance of effects at 3 months posttreatment.

First, for moderate importance in PCS reduction (≥30% reduction in PCS), 62% (31/50) in ER versus 24% (12/51) in WLC met this threshold. For substantial clinical importance in PCS reduction (≥50% reduction in pain), 46% (23/50) in ER versus 12% (6/51) in WLC met this threshold. PCS reductions for ER (mean −8.72, SD 1.03) exceeded the clinically meaningful threshold of 6.8 reported in the literature [78] and is notably greater than that reported in other studies examining in-person 8-week CBT [7,10,79]. Our findings on PCS are expected and aligned with the literature suggesting that pain catastrophizing, an index of pain coping, is highly responsive to behavioral treatments [7,10,79], including SSI [11,12]. For context, multidisciplinary pain rehabilitation research has shown that PCS reductions of 38% are clinically important and associated with less disability and work status at one-year follow-up [80].

Second, for minimal clinical importance in pain reduction (≥15% reduction in pain), 30% (15/50) in ER versus 6% (3/51) in WLC met this threshold. The moderate clinical importance of pain reduction was low in both groups. Importantly, this study used a PROMIS pain measure with a stricter range (5-point scale) versus a traditional 11-point Numeric Pain Rating Scale. As such, the lack of variability in this measure may have negatively impacted our ability to detect the effects of pain intensity.

The tertiary outcomes yielded mixed findings. Reductions in anxiety and depression over time were found in both groups, and the decline was significantly greater for ER than WLC. However, these reductions did not reach the threshold for clinical significance. Notably, for depression, we observed regression to the mean at 3 months with the treatment effect lost, thus suggesting that more intensive treatment might be needed for durable reductions in depressive symptoms. This may be contextualized within widespread social isolation due to COVID-19, which has been shown to worsen depression symptoms over time [81,82], or other stressors related to COVID-19 or the seasonal context of this study.

Interestingly, no treatment or time effects were observed for physical function, only a trend toward significance (*P*=.09), which suggests that the study may be underpowered to detect differences or identify subgroups where effect may exist. In addition, due to a coding error, the short form used in this study did not capture upper extremity function, and scale items focused on lower mobility. As this was a mixed-etiology chronic pain study, comprehensive assessment of physical function is warranted to definitively explore its effects. Exploratory analyses revealed interaction effects for reductions in social isolation, which is a notable and important therapeutic target during the COVID-19 pandemic social restrictions. Although the two groups did not differ at 3 months, the interaction effect revealed a decrease in social isolation in the ER group but not in the WLC group. These findings lend support to ER serving as a buffer against the social threat perpetuated by the pandemic [16,83], which can ameliorate its impact on people’s overall health status [65,66].

Although a growing body of research exists on the efficacy of ER for chronic and acute pain management [12,33,36,52], the use of Zoom as a platform to deliver behavioral medicine for chronic pain, particularly single-session treatment, remains novel and understudied. In addition, the extant literature on digital behavioral health research has reported participant treatment engagement rates ranging from 20% to 60% [33,84-86]. Strikingly, the current trial demonstrated a 94% (47/50) engagement rate in the treatment arm. These results also highlight the public interest in the web-based delivery of behavioral medicine interventions as a home-based chronic pain treatment modality. Combined with high participant engagement data and high satisfaction with using the Zoom platform, these data extend prior work [18-25] supporting the utility, user satisfaction, and efficacy of videoconference-delivered interventions for chronic pain.

### Strengths and Limitations

Several findings should be considered within a number of limitations. First, given that the participant population was on average of higher socioeconomic status, as evidenced by their average education and income levels, the results may not be generalizable to populations of different demographics. No data were available on medical diagnoses, current prescriptions, over-the-counter medication use, or concurrent treatments for either study group. In addition, all data were self-reported, and we did not control for receipt of medical care. In light of COVID-19 restrictions, participants were likely to receive their care via web-based delivery as well. In addition, the pain intensity measure used was a 5-point Numeric Pain Rating Scale, as opposed to the 11-point Numeric Pain Rating Scale [87] typically used in research, limiting variability and may have obscured treatment effects and clinically meaningful findings. Finally, the participants were unblinded to the study group assignment. However, given that ER is low risk and low burden, placebo effects are less concerning. Replication studies were indicated with participants with more demographic variability over a longer follow-up period. Finally, future studies should characterize pain diagnoses and pain types.

Despite these limitations, this web-based study evidenced strong interest and participant engagement with single-session skills-based pain treatment, thus supporting further research and extension of web-based ER into clinical care. Second, the COVID-19 context and web-based delivery modality support the ecological validity of the study findings. Notably, the study was conducted remotely and did not benefit from any in-person contact that occurred when research was conducted in medical treatment settings (ie, halo effects). Additional aspects of methodological rigor included analyst blinding, intention-to-treat analyses, and randomization. Finally, our sample included minimal exclusions, which rendered it highly generalizable to other *real-world* individuals.

This study examined class cohorts comprising approximately 25 participants, and in-person class sizes ranged between 20 and 85, thus illustrating the promise of scalable pain care. Indeed, ER may help address pain disparities by ensuring rapid and more equitable access to pain treatment. Our findings provide initial evidence that the web-based delivery of ER may efficiently reduce the burden of chronic pain and improve symptom management. Additional research is needed to test the pragmatic comparative effectiveness of web-based ER to other treatments, such as gold-standard 8-week CBT, and to determine the heterogeneity of treatment effects.

### Conclusions

In 2019, the US Health and Human Services cited ER as a promising scalable behavioral pain treatment [88]. For the first time, this study determined the preliminary efficacy of the Zoom-delivered class in reducing the burden of chronic pain and improving symptom management. Importantly, the web-based single-session class addresses the rapidly expanding need for alternatives to face-to-face encounters due to the COVID-19 pandemic. Web-based delivery of ER stands to largely improve patient access and engagement because it is adaptable to medical or community settings, is readily extendable to underserved populations (eg, rural locations), and may be offered at a low cost.

## Appendix

Multimedia Appendix 1 Consent form.

Multimedia Appendix 2 CONSORT-eHEALTH checklist (V 1.6.1).

## Abbreviations

CBT: cognitive behavioral therapy
CONSORT: Consolidated Standards of Reporting Trials
ER: empowered relief
HIPAA: Health Insurance Portability and Accountability Act
PCS: pain catastrophizing scale
PROMIS: Patient-Reported Outcomes Measurement Information System
REDCap: Research Electronic Data Capture
SSI: single-session intervention
WLC: waitlist control
