# Supplementary material for: Efficacy of a Single-Session “Empowered Relief” Zoom-Delivered Group Intervention for Chronic Pain: Randomized Controlled Trial Conducted During the COVID-19 Pandemic
Source: J Med Internet Res. 2021 Sep 10;23(9):e29672. doi: 10.2196/29672 (PMC8463950; doi:10.2196/29672)
Supplement: Multimedia Appendix 1 [file jmir_v23i9e29672_app1.doc]

**FOR QUESTIONS ABOUT THE STUDY, CONTACT:** Dr. Beth Darnall, Stanford Systems Neuroscience and Pain Laboratory, 1070 Arastradero, Suite 200, MC 5596, Palo Alto, CA 94304-1336. Phone number is (650) 721-2104.

**DESCRIPTION:** You are invited to participate in a research study investigating how a zoom-based pain education class can help people suffering from chronic pain.

**Prior to Class:**  You will be asked to fill out 1 questionnaire before you attend the class. The questionnaire you will be completing will ask you about your pain, mood, beliefs and questions related to COVID-19.

**Pain Education Class:**

You may be randomly assigned to pain education class or to usual care (waitlist). Both are described below. You cannot be reassigned once you are allocated to a class. Upon completion of the 3-month study, participants in the waitlist will be invited to enroll in the pain education class.

1. Pain education class, “Empowered Relief” is about how you relate to your chronic pain. The class will teach you to develop skills to better manage your pain and to avoid catastrophizing of pain. You will be asked to complete a personalized plan to use the skills you learn. After the class we will ask you to fill out an anonymous questionnaire about your experience. Treatment participants will receive an audio file (Relaxation Response) at the end of the class. We aim to give all patients rapid access to low-risk care that empowers them to self-manage their pain. You will be asked to come to one class session online via Zoom or in person. The class held via Zoom may be recorded for training purposes. The recordings will not disclose any names or images of participants. Participants will be notified prior to the class by a research coordinator if their class will be recorded. The class recordings will be destroyed within 5 years after study completion.
2. Usual Care (waitlist): If you are randomized in the waitlist or usual care you will not receive the pain education class and will continue the care you would normally receive as part of your ongoing clinical care.

**Post-Class:** You will be asked to complete 5 follow-up surveys at 1 & 2 weeks and 1, 2, & 3 months post-class to determine whether the class confers long-term benefits across various aspects of health. These questions may take approximately 15-20 minutes to complete and include questions about your symptoms, mood, and function. This may occur via secure web-survey, regular mail, or by phone interview. In light of the coronavirus pandemic, we are also interested in assessing how COVID-19 has impacted your pain and your psychological health. The COVID-19 specific questions may be asked once before treatment and then on each follow-up surveys.

**RISKS AND BENEFITS:** The risks associated with this study are that you might feel uncomfortable answering some questions on the questionnaires. You have the choice not to answer any question that makes you uncomfortable. There is also the risk of your personal information accidentally being disclosed to people outside the study staff, however we are keeping your information in a secure location and on an encrypted, password protected computer. The responses to questions concerning illegal drug use could be self-incriminating and harmful to you if they became known outside the study, however, we do not intend to disclose this information.

We cannot and do not guarantee or promise that you will receive any benefits from this study. Your participation in the pain education class may help you learn new skills to better cope with your chronic pain. Your decision whether or not to participate in this study will not affect your medical care.

**PARTICIPANT SAFETY:** It is possible that, based on information gained from this study, the researchers may be required to report information (e.g., information relating to suicide, physical or sexual abuse) to the appropriate authorities. It is also possible that, based on information gained from this study, the researchers may have serious concerns (relating to matters such as severe depression, physical abuse, etc.) about your health and/or safety; in such a case, the researchers may contact you and provide a referral for your care.

**TIME INVOLVEMENT:** Your participation in this study will take approximately 3 months from enrollment to completion. Questionnaire completion may take approximately 15-20 minutes at each time point: before and after class (1 & 2 weeks and 1, 2, & 3 months post-class). Overall, you will be dedicating about 3.5 hours of your time, which includes the class and the surveys.

**WITHDRAWAL FROM STUDY**

The Protocol Director may also withdraw you from the study without your consent for one or more of the following reasons:

- - Failure to follow the instructions of the Protocol Director and study staff.
  - The Protocol Director decides that continuing your participation could be harmful to you.
  - You need treatment not allowed in the study.
  - The study is cancelled.
  - Other administrative reasons.
  - Unanticipated circumstances.

**PAYMENTS:**  You may receive up to $50 as payment for your participation in the study, which includes completing the pre-class survey and questionnaires for the entire 3 months post class period. If you will not complete the study, you will only be paid for the surveys which you have completed.

Payments may only be made to U.S. citizens, legal resident aliens, and those who have a work eligible visa. You may need to provide your social security number to receive payments.

**PARTICIPANT’S RIGHTS:** If you have read this form and have decided to participate in this project, please understand your participation is voluntary and you have the right to withdraw your consent or discontinue participation at any time without penalty or loss of benefits to which you are otherwise entitled.

The results of this research study may be presented at scientific or professional meetings or published in scientific journals. However, your identity will not be disclosed**.** You have the right to refuse to answer particular questions.

**Authorization To Use Your Health Information For Research Purposes**

Because information about you and your health is personal and private, it generally cannot be used in this research study without your written authorization. If you sign this form, it will provide that authorization. The form is intended to inform you about how your health information will be used or disclosed in the study. Your information will only be used in accordance with this authorization form and the informed consent form and as required or allowed by law. Please read it carefully before signing it.

**What is the purpose of this research study and how will my health information be utilized in the study?**

The purpose of this study is to see if taking a pain education class on managing pain changes the way you deal with your chronic pain. Information we collect from you will be used to evaluate the effectiveness of the class, and may be used in publications as well.

**Do I have to sign this authorization form?**

You do not have to sign this authorization form. But if you do not, you will not be able to participate in this research study. Signing the form is not a condition for receiving any medical care outside the study.

**If I sign, can I revoke it or withdraw from the research later?**

If you decide to participate, you are free to withdraw your authorization regarding the use and disclosure of your health information (and to discontinue any other participation in the study) at any time. After any revocation, your health information will no longer be used or disclosed in the study, except to the extent that the law allows us to continue using your information (e.g., necessary to maintain integrity of research). If you wish to revoke your authorization for the research use or disclosure of your health information in this study, you must write to: Dr. Beth Darnall or Dr. Maisa Ziadni at 1070 Arastradero, Suite 200, MC 5596, Palo Alto, CA 94304-1336.

**What Personal Information Will Be Obtained, Used or Disclosed?**

Your name, medical record number, contact information, date of birth, demographic information, and health information related to this study, may be used or disclosed in connection with this research study, including, but not limited to, responses to questionnaires, medical history, pain conditions, treatment and medication information, medical visits, and changes in treatment or medications. Additionally, your health information related to this study will be obtained through your medical record and the Stanford Pain Management Center’s registry database ("HERO") - which collects questionnaire responses.

**Who May Use or Disclose the Information?**

The following parties are authorized to use and/or disclose your health information in connection with this research study:

- The Protocol Director, Dr. Beth Darnall
- The Stanford University Administrative Panel on Human Subjects in Medical Research and any other unit of Stanford University as necessary
- Research Staff

**Who May Receive or Use the Information?**

The parties listed in the preceding paragraph may disclose your health information to the following persons and organizations for their use in connection with this research study:

- The Office for Human Research Protections in the U.S. Department of Health and Human Services

Your information may be re-disclosed by the recipients described above, if they are not required by law to protect the privacy of the information.

**When will my authorization expire?**

Your authorization for the use and/or disclosure of your health information will end on December 31^st^, 2050 or when the research project ends, whichever is earlier.

**Contact Information:**
If you have any questions, concerns or complaints about this research study, its procedures, risks and benefits, or alternative courses of treatment, you should ask the Protocol Director, Dr. Beth Darnall at (650) 721-2104.  You should also contact her at any time if you feel you have been hurt by being a part of this study.

Independent Contact: If you are not satisfied with how this study is being conducted, or if you have any concerns, complaints, or general questions about the research or your rights as a participant, please contact the Stanford Institutional Review Board (IRB) to speak to someone independent of the research team at (650)-723-5244 or toll free at 1-866-680-2906.  You can also write to the Stanford IRB, Stanford University, 1705 El Camino Real, Palo Alto, CA 94306.
